# Serum hepatitis B core antibody as a biomarker of hepatic inflammation in chronic hepatitis B patients with normal alanine aminotransferase

**DOI:** 10.1038/s41598-017-03102-3

**Published:** 2017-06-05

**Authors:** Jiyuan Zhou, Liuwei Song, Hong Zhao, Linlin Yan, Anlin Ma, Shibin Xie, Xuqing Zhang, Dazhi Zhang, Qing Xie, Guo Zhang, Jia Shang, Jun Cheng, Weifeng Zhao, Zhiqiang Zou, Mingxiang Zhang, Ningshao Xia, Guiqiang Wang

**Affiliations:** 1 Department of Infectious Disease, Center for Liver Disease, Peking University First Hospital, Beijing, China; 2 State Key Laboratory of Molecular Vaccinology and Molecular Diagnostics, National Institute of Diagnostics and Vaccine Development in Infectious Diseases, School of Public Health, Xiamen University, Xiamen, China; 3 Department of Infectious Disease, China-Japan Friendship Hospital, Beijing, China; 4 Department of Infectious Disease, The Third Affiliated Hospital Sun Yat-Sen University, Guangzhou, Guangdong China; 5 Department of Infectious Diseases, South West Hospital affiliated to Third Military Medical University, Chongqing, China; 6 Department of Infectious Diseases, Second Affiliated Hospital of Chongqing Medical University, Chongqing, China; 7 Department of Infectious Diseases, Rui Jin Hospital Shanghai Jiao Tong University School of Medicine, Shanghai, China; 8 Department of Infectious Diseases, The People’s Hospital of Guang Xi Zhuang Autonomous Region, Nanning, Guangxi China; 9 Department of Infectious Diseases, The People’s Hospital of He Nan Province, Zhengzhou, Henan China; 10 Department of Infectious Diseases, Di Tan Hospital affiliated to Capital Medical University, Beijing, China; 11 Department of Infectious Diseases, Xinxiang Medical University Third Hospital, Xinxiang, Henan China; 12 Department of Infectious Diseases, Yan tai City Hospital for Infectious Disease, Yan tai, Shandong China; 13 Department of Infectious Diseases, Shenyang Sixth People’s Hospital, Shenyang, Liaoning China; 14 Collaborative Innovation Center for Diagnosis and Treatment of Infectious Diseases, Zhejiang University, Hangzhou, Zhejiang China

## Abstract

Our previous studies unexpectedly indicated that the level of serum hepatitis B core antibody (anti-HBc) was positively correlated with the serum alanine aminotransferase (ALT) level. The aim of this study was to determine whether anti-HBc could serve as a potential biomarker for the detection of liver inflammation in chronic hepatitis B (CHB) patients, especially in patients with normal ALT levels. Serum anti-HBc levels were quantified in 655 treatment-naïve CHB patients, including 45 patients who underwent two liver biopsies (baseline phase and the 78^th^ weeks of antiviral-treatment). Serum anti-HBc levels increased significantly along with the increasing histology activity index (HAI) score. After antiviral-treatment, patients with HAI score reduction had significant decline in serum anti-HBc level. Multivariate analysis showed that anti-HBc was independently associated with moderate-to-severe hepatic inflammation in patients with normal ALT level. Furthermore, serum anti-HBc showed a high diagnostic accuracy for predicting moderate-to-severe inflammation in both hepatitis B e antigen (HBeAg)-positive and HBeAg-negative CHB patients with normal ALT levels (area under the curve, AUC = 0.87 and 0.75; respectively). Thus, anti-HBc may be a strong indicator for assessing the hepatic inflammatory degree and used for antiviral treatment decisions in CHB patients with normal ALT levels.

## Introduction

Hepatitis B virus (HBV) infection is the most common chronic viral infection, and is the major cause of hepatocellular carcinoma (HCC), one of the most frequent cancers in Asian-Pacific region especially in China^1, 2^.

HBV is not cytopathogenic and is indirectly involved in the occurrence of hepatocyte damage and necroinflammation by immune-mediated responses^3^. In the progression of chronic hepatitis B (CHB), the persistent inflammation burden of the liver is not only the main risk factor for the development of liver cirrhosis and hepatocellular carcinoma (HCC), but it also leads to ineffective HBV clearance^3–5^. Clinically, serum alanine aminotransferase (ALT) has been widely used for evaluating the severity of hepatic inflammation in liver disease. However, numerous studies have reported that some CHB patients with normal ALT levels have severe liver damage and require antivirus treatment according to current guidelines^6, 7^. Therefore, the accurate assessment and monitoring of the severity of liver inflammation plays an important role not only in the control of disease progression, but in the therapy decision for patients with normal ALT levels.

Recently, quantitative antibodies to hepatitis B core antigen (anti-HBc) levels have been reported to predict the treatment response for CHB patients receiving antiviral therapies^8–10^. Patients with high baseline anti-HBc levels had a significantly higher response than patients with low baseline anti-HBc level. Furthermore, our previous studies also suggested that the anti-HBc level was positively correlated with ALT and that the proposed serum anti-HBc level could be a potential biomarker for hepatic inflammatory activity in CHB patients^9^. However, no direct evidence has reported.

Therefore, we aimed to determine whether serum anti-HBc could serve as a potential biomarker for the detection of the severity of liver inflammation, and used for antiviral treatment decisions CHB patients with normal ALT levels.

## Results

### CHB patients’ characteristics

655 CHB patients were enrolled in the cohort study, which included 404 hepatitis B e antigen (HBeAg) -positive (HBeAg [+]) and 251 HBeAg-negative (HBeAg [+]) CHB patients. In 98 HBeAg (+) patients with normal ALT levels, 35 (35.7%) patients had at least moderate inflammation, and 33 (33.7%) had significant fibrosis. In 95 HBeAg (−) patients with normal ALT levels, 35 (36.8%) patients had at least moderate inflammation, and 30 (31.6%) had significant fibrosis. The CHB patient’s characteristics at the time of liver biopsy are summarized in Table 1.

**Table 1:** Clinical characteristics of patients with chronic hepatitis B virus infection.

Parameters	HBeAg (+) Patients (n = 404)			HBeAg (−) Patients (251)		
	Normal ALT (n = 98)	ALT > ULN (n = 306)	*P* value	Normal ALT (n = 95)	ALT > ULN (n = 156)	*P* value
Sex distribution, males	75.5% (74/98)	80.8% (248/306)	0.25	63.2% (60/95)	84% (131/156)	<0.001
age, years	37.56 ± 10.07	35.90 ± 10.4	0.14	42.26 ± 10.61	41.25 ± 8.98	0.37
BMI (kg/m^2^)	22.7 ± 2.54	24.31 ± 16.69	0.12	23.87 ± 3.04	23.49 ± 2.71	0.23
HBsAg (log_10_ IU/ml)	3.86 ± 0.95	3.82 ± 0.76	0.42	3.17 ± 0.65	3.15 ± 0.77	0.99
HBV DNA (log_10_ IU/ml)	6.78 ± 1.69	6.97 ± 1.65	0.35	4.43 ± 1.61	5.09 ± 1.61	0.002
Anti-HBc (log_10_ IU/ml)	3.94 ± 1.09	4.41 ± 0.82	<0.001	4.30 ± 0.65	4.58 ± 0.55	<0.001
ALT (U/L)	27.35 ± 8.36	131.4 ± 151.4	<0.001	27.53 ± 8.59	112.8 ± 122.8	<0.001
AST (U/L)	28 ± 13.76	77.6 ± 86.5	<0.001	27.50 ± 10.92	74.32 ± 83.46	<0.001
ALP (U/L)	69.34 ± 22.33	84.68 ± 28.18	<0.001	76.79 ± 23.95	86.34 ± 30.71	0.03
GGT (U/L)	30.42 ± 31.18	62.27 ± 63.20	<0.001	33.15 ± 33.92	64.76 ± 62.30	<0.001
Tbil	14.42 ± 7.01	17.36 ± 17.34	0.15	19.48 ± 41.59	17.87 ± 11.82	0.07
Albumin (g/L)	44.04 ± 4.39	43.64 ± 5.36	0.28	44.89 ± 5.39	44.92 ± 6.44	0.41
PT s	12.76 ± 1.65	12.81 ± 1.45	0.47	12.33 ± 1.41	12.68 ± 1.52	0.08
PTA %	98.66 ± 19.58	96.60 ± 16.64	0.58	98.97 ± 14.89	95.30 ± 17.96	0.2
INR	1.04 ± 0.15	1.04 ± 0.12	0.42	1 ± 0.09	1.04 ± 1.11	0.03
Platelet counts (x10^9^/L)	186.5 ± 57.61	176.3 ± 55.37	0.17	167.7 ± 52.46	153.6 ± 56.45	0.04
Histology (n, %)						
HAI Score 0–4	64.3% (63/98)	30.1% (92/306)	<0.001	63.2% (60/95)	32.7% (51/156)	<0.001
≥5	35.7% (35/98)	69.9% (214/306)		36.8% (35/95)	67.3% (105/156)	
Fibrosis Score 0–2	66.3% (65/98)	62.4% (191/306)	0.55	68.4% (65/95)	53.8% (84/156)	0.02
≥3	33.7% (33/98)	37.6% (115/306)		31.6% (30/95)	46.2% (72/156)	

MBI, body mass index; HBsAg, hepatitis B surface antigen; HBeAg, hepatitis B e antigen; anti-HBc, hepatitis B core antibody; ALT, alanine aminotransferase; AST, aspartate aminotransferase; ALP, alkaline phosphatase; GGT, gamma-glutamyl transpeptidase; Tbil, total bilirubin; PT, prothrombin time; PTA, prothrombin time activity; INR, international normalized ratio; HAI, histology activity index.

### Significantly increased levels of serum anti-HBc correlate with histological inflammation in CHB patients

**Figure 1:**
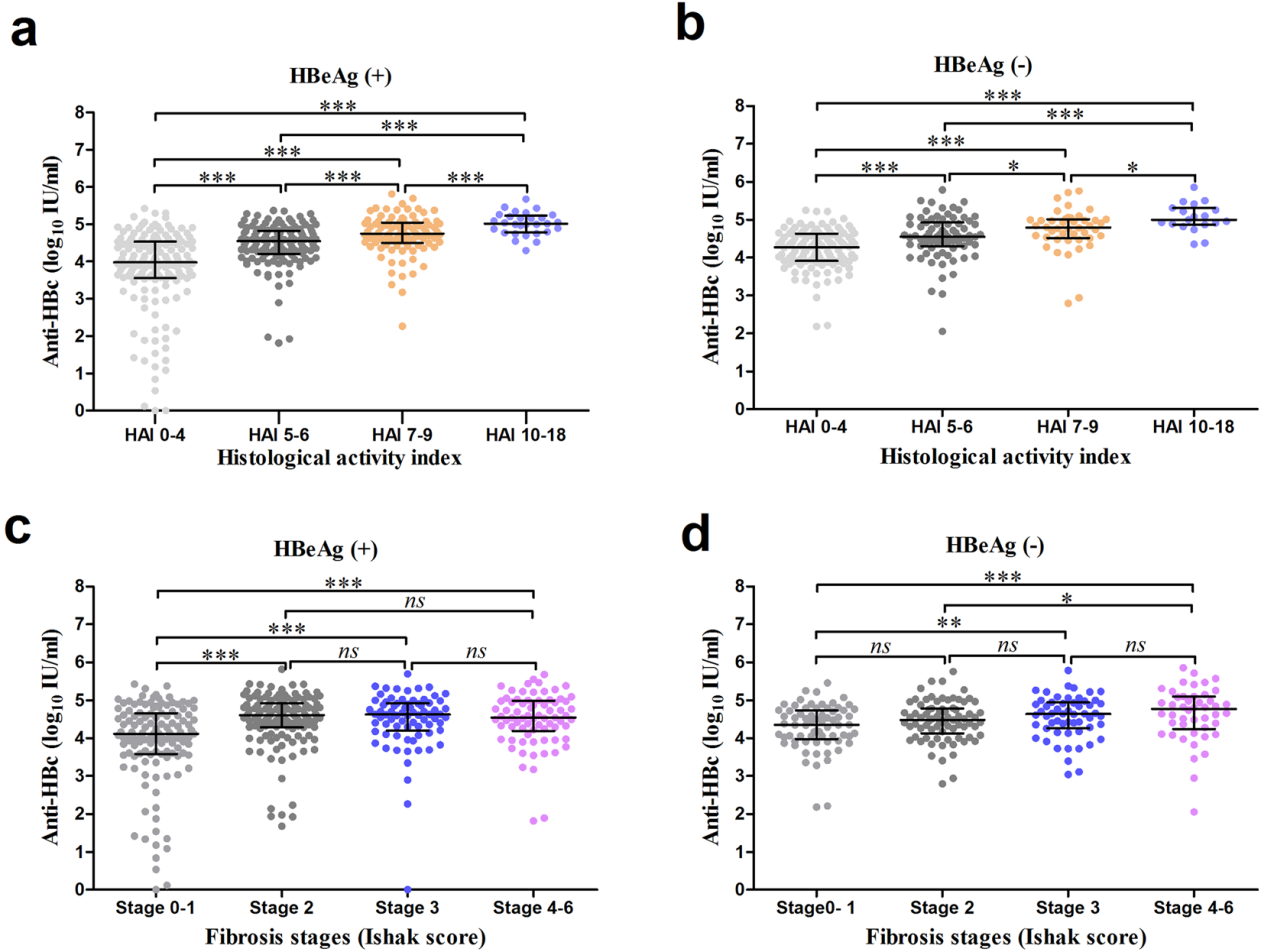
Serum anti-HBc level increased significantly along with increasing histology activity index score. Serum anti-HBc level in different stages of histology activity index score in total HBeAg (+) (**a**) and HBeAg (−) (**b**) CHB patients. Serum anti-HBc collected from patients with different stages of CHB-related liver inflammation was quantified for the levels of anti-HBc using ELISA. Graph showing correlation between serum anti-HBc level and stages of liver fibrosis HBeAg (+) (**c**) and HBeAg (−) (**d**) CHB patients. Interquartile ranges with medians presented. ***P < 0.001, **P < 0.01, *P < 0.05 and *ns*, no significance.

In the HBeAg (+) CHB patients, serum anti-HBc levels increased significantly along with the increasing histology activity index (HAI) score (mean ± SD; HAI 0–4: 3.80 ± 1.10 vs. HAI 5–6: 4.45 ± 0.60 vs. HAI 7–9: 4.71 ± 0.55 vs. HAI 10–18: 5.02 ± 0.30, *P* < 0.001 by ANOVA) (Fig. 1a). In the HBeAg (−) CHB patients, serum anti-HBc levels also differed significantly between HAI score (HAI 0–4: 4.21 ± 0.53 vs. HAI 5–6: 4.54 ± 0.60, *P* < 0.001; HAI 5–6 vs. HAI 7–9: 4.74 ± 0.56, *P* < 0.05; HAI 7–9 vs. HAI 10–18: 5.06 ± 0.37, *P* < 0.05) (Fig. 1b). Spearman’s correlation analysis indicated that serum anti-HBc levels were positively correlated with the HAI score in both HBeAg (+) and HBeAg (−) CHB patients (r = 0.549 and 0.494, respectively, *P* < 0.001).

Serum anti-HBc were significantly correlated with liver fibrosis stage in both HBeAg (+) and HBeAg (−) patients (r = 0.233 and 0.240, respectively, *P* < 0.001), whereas there was no significant difference in the anti-HBc level between fibrosis stage score more than 2 (Fig. 1c,d).

### The level of serum anti-HBc decreased along with alleviated histological inflammation in CHB patients receiving antivirus treatment

**Figure 2:**
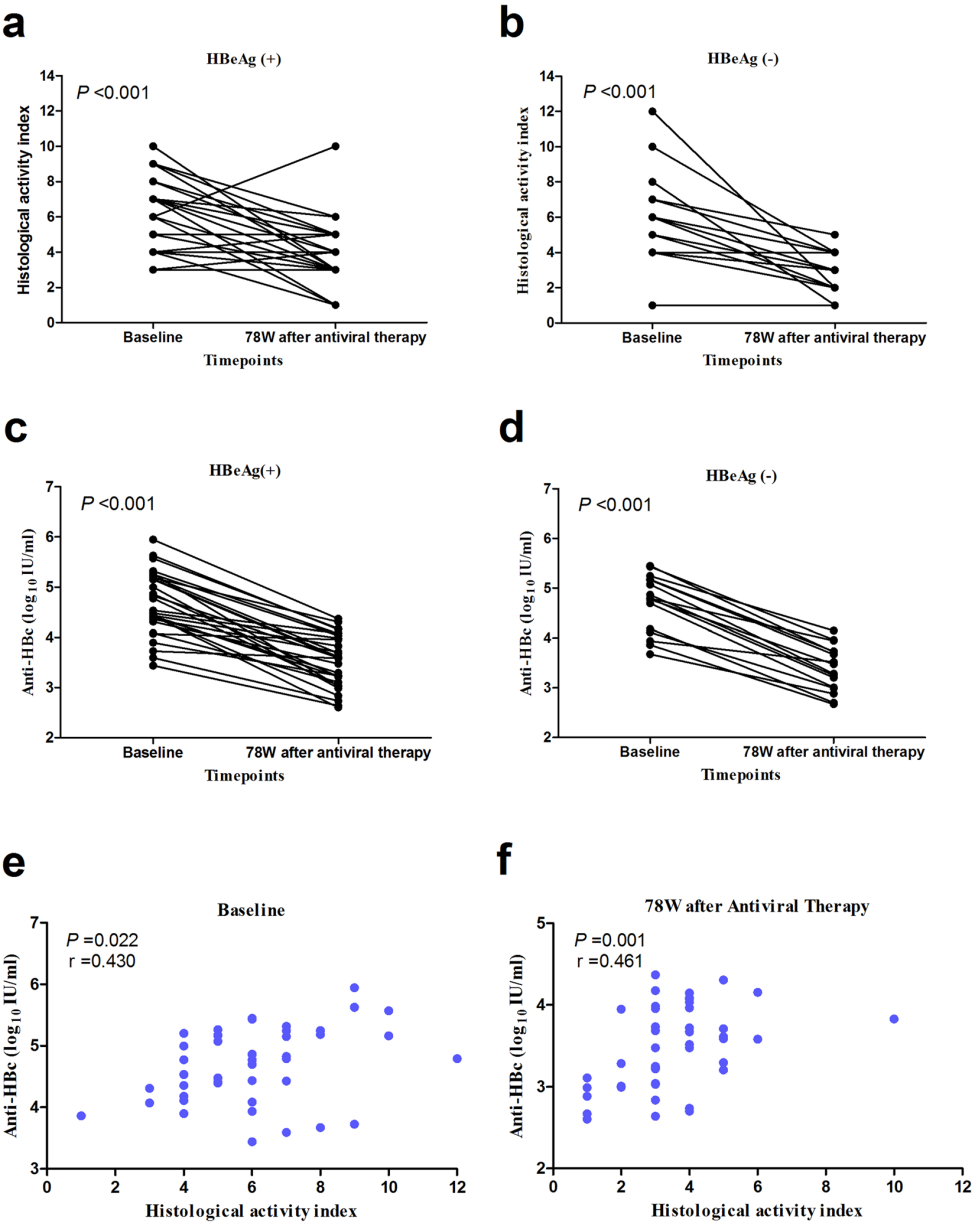
The level of serum anti-HBc decreased along with alleviated histological inflammation in CHB patients receiving antivirus treatment. Dynamic changes of histological activity index score in HBeAg (+) (**a**) and HBeAg (−) (**b**) CHB patients receiving antiviral treatment after a second liver biopsy. Dynamic changes of serum anti-HBc levels in HBeAg (+) (**c**) and HBeAg (−) (**d**) CHB patients receiving antiviral treatment after a second liver biopsy. The correlation between serum anti-HBc levels and HAI score in the baseline (**e**) and 78th week time point (**f**).

To evaluate the correlation between anti-HBc and the severity of liver inflammation in a longitudinal cohort, of the aforementioned 655 patients, 45 CHB patients (29 HBeAg [+] and 16 HBeAg [−]) undergoing second liver biopsies after 78 weeks of antiviral-treatment were further analyzed. The levels of serum anti-HBc decreased in accordance with the alleviated histological inflammation, and the levels were significantly different between the baseline and 78^th^ week time point (*P* < 0.001, Fig. 2a–d). However, in this study, the fibrosis score remained stable after 78 weeks antiviral therapy (Supplementary Figure S1).

As shown in Fig. 2e–f, there was a significant correlation of between the anti-HBc level and HAI score both in the first liver biopsy (baseline) and the second liver biopsy (78^th^ week) (r = 0.430 *P* = 0.022 and r = 0.461 *P* = 0.001; respectively), suggested that serum anti-HBc levels were positively correlated with severity of hepatic inflammation in the longitudinal cohort study.

### Serum anti-HBc was better than ALT in correlation with histological inflammation in CHB patients

Clinically, ALT as a noninvasive marker has been widely used for detecting the severity of hepatic inflammation in liver disease. To compare anti-HBc to ALT, the correlation between ALT and HAI score was further analyzed. In both HBeAg (+) and HBeAg (−) patients, serum ALT levels were significantly correlated with HAI score in patients with ALT >2 × ULN (r = 0.207 *P* = 0.01 and 0.311 *P* < 0.01; respectively), whereas that was no significance in patients with normal ALT levels (r = 0.126 *P* = 0.21 and r = 0.156 *P* = 0.13; respectively) and patients with 1 × ULN < ALT ≤ 2 × ULN (r = 0.122 *P* = 0.12 and r = 0.080 *P* = 0.46; respectively). Nevertheless, in both HBeAg (+) and HBeAg (−) CHB patients, the correlation between serum anti-HBc and HAI score in patients with normal ALT levels (r = 0.617 and 0.378, respectively; all *P* < 0.001) and patient with ULN < ALT ≤ 2 × ULN (r = 0.427 and 0.428, respectively; all *P* < 0.001) were much better than that in patients with ALT >2 × ULN (r = 0.263 *P* < 0.01 and 0.232 *P* = 0.06; respectively) (Table 2). Therefore, serum anti-HBc could be better than ALT in correlation with HAI score in patients with ALT ≤2 × ULN.

**Table 2:** Correlation between histology activity index score and anti-HBc levels, ALT levels according to the ALT stratum.

	Correction	ALT stratum (*Spearman’r; P* value*)*		
		ALT < 1 × ULN	1 × ULN < ALT < 2 × ULN	ALT > 2 × ULN
HBeAg + (404)	ALT vs. anti-HBc	0.044; 0.66	0.138; 0.08	0.250; **<**0.01
	ALT vs. HAI	0.126; 0.21	0.122; 0.12	0.207; 0.01
	anti-HBc vs. HAI	0.617; **<**0.001	0.427; **<**0.001	0.263; **<**0.01
HBeAg− (251)	ALT vs. anti-HBc	0.191; 0.06	0.195; 0.06	0.219; 0.03
	ALT vs. HAI	0.156; 0.13	0.080; 0.46	0.311; <0.01
	anti-HBc vs. HAI	0.378; **<**0.001	0.428; **<**0.001	0.232; 0.06

ALT, alanine aminotransferase; HBeAg, hepatitis B e antigen ULN, upper limit of normal; HAI, histology activity index.

### Serum anti-HBc can significantly differentiate between mild or no inflammation and moderate-to-severe inflammation in patients with normal ALT levels

Patients with moderate- to-severe inflammation had significantly higher levels of HBsAg, HBV DNA, anti-HBc, ALT, AST, ALP, GGT and Albumin than the patients with mild or no inflammation. Multivariate analysis indicated that anti-HBc was independently associated with moderate-to-severe inflammation in both HBeAg (+) and HBeAg (−) CHB patients (Supplementary Tables S2 and 3). However, in this study, anti-HBc was not independently associated with significant fibrosis in CHB patients by univariate and multivariate analysis (Supplementary Tables S4 and 5).

**Figure 3:**
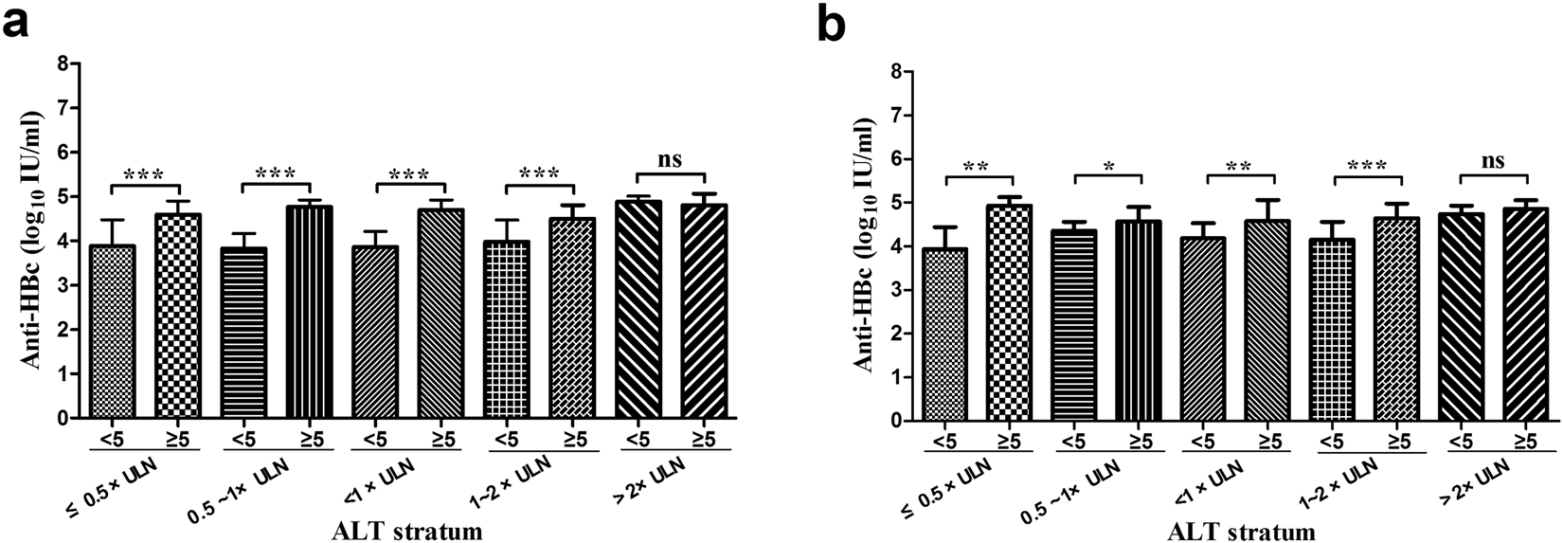
Correlation between Anti-HBc levels and histological scores of activity in HBeAg (+) (**a**) and HBeAg (−) (**b**), according to the ALT stratum. ***P < 0.001, **P < 0.01, *P < 0.05 and *ns*, no significance. Interquartile ranges with medians presented. ALT, alanine aminotransferase; ULN, upper limit of normal; HAI, histology activity index.

Furthermore, we further divided the recruited patients into the following four groups based on the Asian-Pacific consensus statement on the management of chronic hepatitis B: Low normal group, ALT ≤0.5 × ULN; High normal group, ALT 0.5–1 × ULN; Minimally raised group, ALT 1–2 × ULN and raised groups, ALT >2 × ULN^4^. As shown in Fig. 3, apart from raised groups, patients with moderate-to-severe inflammation had significantly higher levels of serum anti-HBc than the patients with mild or no inflammation in all groups. Altogether, these results indicate that serum anti-HBc as a potential biomarker could be used for differentiating between mild or no inflammation and moderate-to-severe inflammation in patients with ALT ≤2 × ULN, especially in patients with normal ALT levels.

### Serum anti-HBc was an independent predictors of moderate-to-severe inflammation in patents with normal ALT levels

To investigate whether anti-HBc is independently associated with moderate-to-severe inflammation in patents with normal ALT levels, the univariate and multivariate analysis of clinical parameters, anti-HBc with liver inflammation was further analyzed. In HBeAg (+) CHB patients, the serum levels of HBsAg, HBV DNA, anti-HBc, AST, GGT, TBil and platelet counts were significantly correlated with moderate-to-severe inflammation based on the univariate analysis, whereas the multivariate logistic regression analysis indicated that the anti-HBc (*P* = 0.009, odd ratio = 4.78), HBV DNA (*P* = 0.04, odd ratio = 0.64) and TBil (*P* = 0.03, odd ratio = 1.02) could be used to independently predict moderate-to-severe inflammation (Table 3).

**Table 3:** Univariate and multivariate analysis of clinical parameters, cytokines with histological activity index in HBeAg (+) patients with normal ALT levels (n = 98).

Parameters	Univariate analysis		*P* Value (Univariate)	Multivariate analysis	*P* Value (Multivariate)
	HAI < 5 (n = 63)	HAI ≥ 5 (n = 35)		Odds Ratio (95% CI)	
Sex distribution, males	74.6% (47/63)	77.14% (27/35)	1		
age, years	37.11 ± 10.38	38.37 ± 9.58	0.39		
BMI (kg/m^2^)	22.86 ± 2.64	22.40 ± 2.34	0.47		
HBsAg (log_10_ IU/ml)	4.05 ± 1.05	3.51 ± 0.61	<0.001	1.27 (0.59–2.76)	0.54
HBV DNA (log_10_ IU/ml)	7.16 ± 1.60	6.12 ± 1.66	0.001	0.64 (0.42–0.98)	0.04
Anti-HBc (log_10_ IU/ml)	3.89 ± 0.89	4.44 ± 0.71	<0.001	4.78 (1.48–15.43)	0.009
ALT (U/L)	26.92 ± 7.98	28.13 ± 9.07	0.34		
AST (U/L)	24.10 ± 6.25	35.23 ± 19.86	<0.001	1.08 (1.00–1.17)	0.33
ALP (U/L)	67.59 ± 20.17	72.67 ± 26.00	0.65		
GGT (U/L)	23.77 ± 16.37	42.64 ± 45.63	0.003	1.02 (0.99–1.06)	0.56
Tbil	12.52 ± 4.57	17.93 ± 9.17	<0.001	1.02 (0.99–1.07)	0.03
Albumin	44.34 ± 4.02	43.47 ± 5.01	0.36		
PT s	12.74 ± 0.96	12.82 ± 2.48	0.49		
PTA %	100.2 ± 19.76	95.88 ± 19.23	0.67		
INR	1.007 ± 0.089	1.06 ± 0.20	0.47		
Platelet counts (x10^9^/L)	199.3 ± 54.26	163.3 ± 56.93	0.003	1.00 (0.99–1.01)	0.78

MBI, body mass index; HBsAg, hepatitis B surface antigen; HBeAg, hepatitis B e antigen; anti-HBc, hepatitis B core antibody; ALT, alanine aminotransferase; AST, aspartate aminotransferase; ALP, alkaline phosphatase; GGT, gamma-glutamyl transpeptidase; Tbil, total bilirubin; PT, prothrombin time; PTA, prothrombin time activity; INR, international normalized ratio.

In HBeAg (−) CHB patients, the univariate analysis identified anti-HBc, AST and GGT were significantly associated with moderate-to-severe inflammation. The multivariate analysis indicated that only anti-HBc (*P* < 0.001, odd ratio = 5.40) was an independent predictors of moderate-to-severe inflammation (Table 4).

**Table 4:** Univariate and multivariate analysis of clinical parameters, cytokines with histological activity index in HBeAg (−) patients with normal ALT levels (n = 95).

**Parameters**	**Univariate analysis**		***P*** **Value (Univariate)**	**Multivariate analysis**	***P*** **Value (Multivariate)**
	**HAI < 5 (n = 60)**	**HAI ≥ 5 (n = 35)**		**Odds Ratio (95% CI)**	
Sex distribution, males	65% (39/60)	60% (21/35)	0.66		
age, years	39.68 ± 11.54	37.89 ± 9.23	0.64		
BMI (kg/m^2^)	22.95 ± 2.62	22.43 ± 2.54	0.24		
HBsAg (log_10_ IU/ml)	3.66 ± 0.92	3.43 ± 0.57	0.33		
HBV DNA (log_10_ IU/ml)	4.34 ± 1.33	4.57 ± 2.02	0.58		
Anti-HBc (log_10_ IU/ml)	3.81 ± 1.02	4.79 ± 0.64	<0.001	5.40 (1.91–15.29)	<0.001
ALT (U/L)	26.78 ± 8.22	28.8 ± 9.17	0.2		
AST (U/L)	25.65 ± 10.35	30.67 ± 11.29	0.004	1.01 (0.99–1.09)	0.13
ALP (U/L)	75.07 ± 26.11	79.85 ± 24.29	0.35		
GGT (U/L)	26.74 ± 27.54	44.61 ± 41.07	<0.001	1.01 (0.99–1.03)	0.45
Tbil	15.16 ± 7.38	26.88 ± 67.82	0.84		
Albumin	45.21 ± 4.54	44.34 ± 6.66	0.21		
PT s	12.36 ± 1.41	12.29 ± 1.45	0.62		
PTA %	99.59 ± 15.91	97.94 ± 13.19	0.93		
INR	1.00 ± 0.09	1.02 ± 0.10	0.63		
Platelet counts (x10^9^/L)	172.2 ± 48.31	160.0 ± 58.75	0.07		

MBI, body mass index; HBsAg, hepatitis B surface antigen; HBeAg, hepatitis B e antigen; anti-HBc, hepatitis B core antibody; ALT, alanine aminotransferase; AST, aspartate aminotransferase; ALP, alkaline phosphatase; GGT, gamma-glutamyl transpeptidase; Tbil, total bilirubin; PT, prothrombin time; PTA, prothrombin time activity; INR, international normalized ratio.

### Serum anti-HBc holds a better predictive value for differentiating between mild or no and moderate-to-severe in patients with normal ALT levels

**Figure 4:**
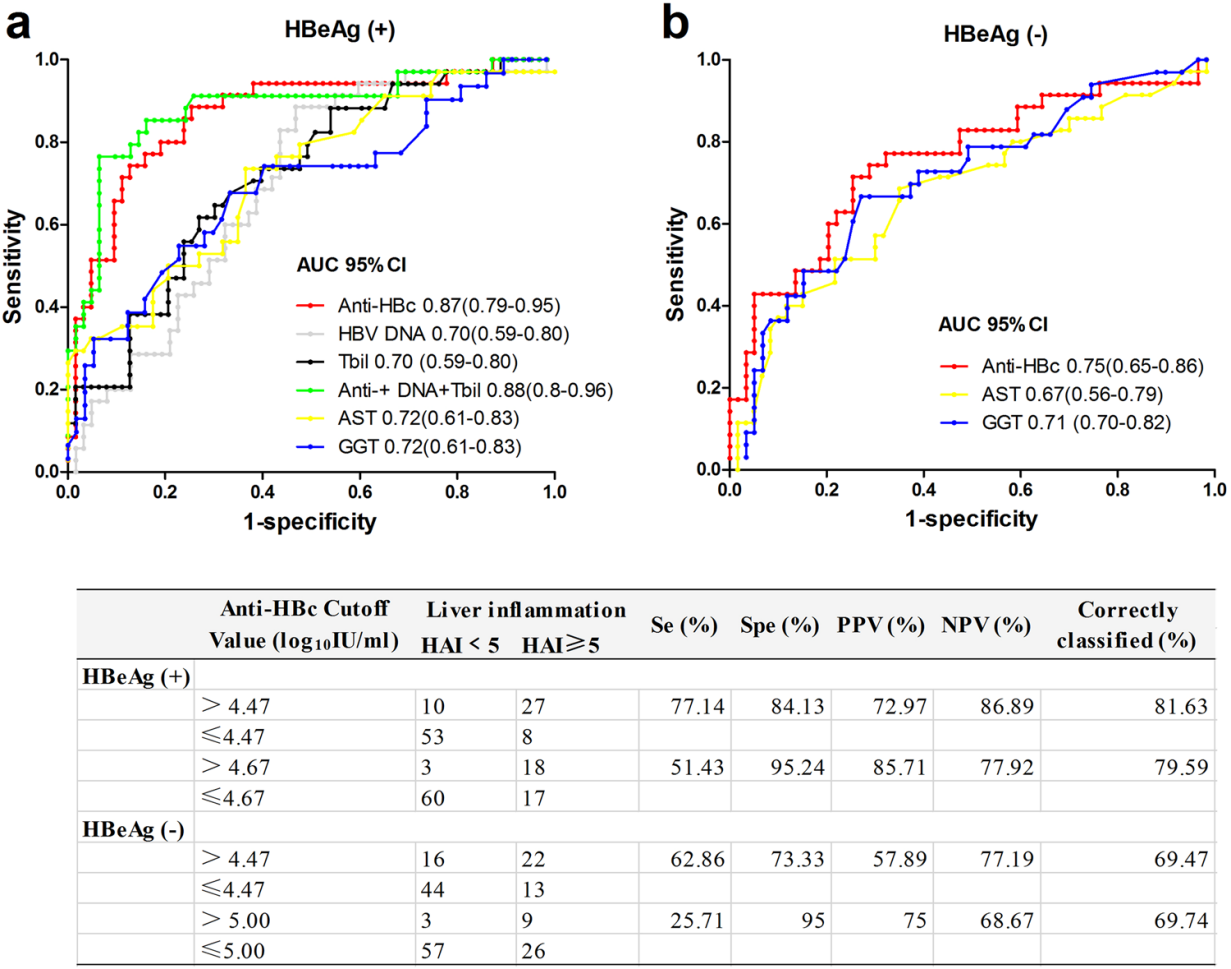
Serum anti-HBc holds a better diagnostic value for differentiating between mild or no (HAI < 5) and moderate-to-severe (HAI ≥ 5) in HBeAg (+) (**a**) and HBeAg (−) (**b**) patents with normal ALT. AUROC, area under receiver operating characteristics curve; GGT, gamma glutamyl transpeptidase; AST, aspartate aminotransferase; Se, sensitivity; Spe, specificity; PPV, positive predictive value; NPV, negative predictive value.

ROC curves were created to evaluate the diagnostic efficiency of serum anti-HBc for differentiating between mild or no inflammation and moderate-to-severe inflammation in CHB patients with normal ALT levels. As shown in Fig. 4, the diagnostic value of anti-HBc was assessed. In HBeAg (+) CHB patients, the AUC of anti-HBc for the prediction of moderate-to-severe inflammation was 0.87. Maximizing the sum of sensitivity and specificity, the optimal cut-offs of anti-HBc was 4.47 log_10_ IU/mL. According to obtaining a specificity of at least 95%, the cut-offs of anti-HBc was 4.67 log_10_ IU/mL. In HBeAg (−) CHB patients, the AUC of anti-HBc for the prediction of moderate-to-severe inflammation was 0.75. The optimal cut-offs of anti-HBc was 4.47 log_10_ IU/mL with a maximizing the sum of sensitivity and specificity and 5.00 log_10_ IU/mL with a specificity of 95%.

It is worth noting that the AUCs of AST and GGT were significantly less effective than anti-HBc for differentiating mild or no inflammation from moderate-to-severe inflammation. Furthermore, the combination index was constructed to explore whether considering HBV DNA and TBil to anti-HBc levels in addition to anti-HBc would lead to a better diagnosis of HBeAg (+) CHB patients with moderate-to-severe inflammation using binary logistic regression. The best formula for predicting moderate-to-severe inflammation was (2.081 × log_10_Anti-HBc IU/ml) − (0.345 × log_10_HBV DNA IU/ml) + (0.134 × TBil IU/ml) −9.008. However, the addition these variables to anti-HBc did not improve its diagnostic efficiency.

## Discussion

To our knowledge, this is the first study to evaluate the serum anti-HBc as a strong indicator for assessing the hepatic inflammatory degree in CHB patients with normal ALT levels. The results of these analyses are supported by the large, multicentral cohorts and strict pathological assessments. The results indicated that the levels of anti-HBc increased significantly along with the increasing HAI score in patients with normal ALT levels. In addition, compared to other predictors, the diagnostic performance of serum anti-HBc for the differentiating between mild or no inflammation and moderate-to-severe inflammation was excellent in both HBeAg (+) and HBeAg (−) CHB patients with normal ALT levels (AUC = 0.87 and 0.75; respectively).

Active significant hepatic inflammation is the main risk factor for developing cirrhosis and HCC in CHB patients^2, 4^. The latest guidelines suggested that early control of developing liver inflammation should be the priority for the detection of liver fibrosis^11^. Thus, accurate evaluation of the initial stage of liver inflammation and progression over time represents a high priority and growing medical need. To date, percutaneous liver biopsy followed by histological analysis remains the gold standard for evaluating the severity of liver injury. Because it is a costly and invasive procedures with inherent risks, the clinical use of this diagnostic method has been restricted^12, 13^. Over the past decades, several non-invasive methods have been developed or are being validated, especially the diagnosis of liver fibrosis^14, 15^. However, there have been few novel markers for assessing the severity of liver inflammation. In clinical practice, serum ALT is an easily accessible surrogate parameter and is commonly used for monitoring the state of liver inflammation^16^. The present study indicated that approximately 36.6% (70/193) of CHB patients with normal ALT levels have moderate-to-severe hepatic inflammation (Table 1), which is higher than the previous reports^4, 6^. For these patients, recent guidelines recommend that CHB patients with moderate-to-severe hepatic inflammation (HAI ≥5) should be considered for antiviral therapy immediately^4, 11^. Thus, use of ALT could be weakness in assessing liver injury and may underestimates the proportion of patients who urgently need antiviral therapy in patients with normal ALT levels. Recent studies have noted the possibility of lowering ULN to 30 IU/L for men and 19 IU/L for women^17, 18^. However, the majority of countries in Asia including China, still use an ALT of 40 IU/ml as ULN^4, 19^. The results of our study may provide support for decreasing the ULN of ALT in Chinese patients, and reducing the portion of patients with moderate-to-severe hepatic inflammation.

Anti-HBc is one of the most universal serological markers for HBV infection and can generally persist throughout a patient’s life, regardless of acute, chronic or past HBV infections^20, 21^. In this study, serum anti-HBc levels were not only correlated positively with the severity of liver inflammation in baseline of total patients, but decreased along with alleviated histological inflammation in CHB patients receiving antivirus treatment. Furtherly, we analyzed the correlation between HAI score and serum anti-HBc, ALT in CHB patients, and the results indicated that serum anti-HBc was better than ALT in correlation with severity of liver inflammation in patients with ALT ≤2 × ULN, especially in patients with normal ALT levels (Table 2). In addition, anti-HBc were significantly different between moderate-to-severe inflammation and no mild inflammation in different groups from patients with normal ALT level. Thus, based on the aforementioned results, we hypothesized that the level of serum anti-HBc could be a potential biomarker for accurate assessment of the severity of liver inflammation in patients with normal ALT levels.

In the present study, we are first to report that increasing levels of serum anti-HBc were independently associated with moderate-to-severe hepatic inflammation in both HBeAg (+) and HBeAg (−) CHB patients with normal ALT levels (odd ratio = 4.78 *P* = 0.009 and odd ratio = 5.40 *P* < 0.001; respectively) based on the multivariate analysis (Tables 3 and 4). The possible mechanism underlying the roles of anti-HBc in hepatic inflammation throughout the progression of chronic HBV infection is still unknown. Chronic HBV infection involves different cells of the innate and adaptive immune responses^1^. During a cellular immune response, the T lymphocytes subgroups such as CD4^+^ and CD8^+^ cells respond to this virus and can directly cause the persistent inflammation of chronic hepatitis B^1^. However, the role of B-lymphocytes in the severity of chronic hepatitis B is unclear^22^. A recent study reported that high prevalence of activated B cells plays a crucial role in the progression of CHB infection by B lymphocytes-mediated immune response to HBV^23^. Hepatitis B core protein (HBcAg) is the most immunogenic HBV antigen, and its antibody, anti-HBc secreted via the activated antibody-secreting B cells directly play an important role in the severity of chronic hepatitis B by hepatocytotoxic response^22^. Furthermore, Li *et al*. found that circulating CXCR5^+^CD4^+^ T cells can help naïve B cells to produce anti-HBc via IL-21 in patients with chronic HBV infection^24^. The percentage of circulating CXCR5^+^CD4^+^ T cells was positively correlated with serum levels of ALT and AST, suggesting that the frequency and phenotype of CXCR5+CD4+ T cells is associated with HBV-related liver damage^25^. Therefore, we hypothesized that the anti-HBc could play an important role in liver inflammation of CHB patients through the hepatocytotoxic effects of anti-HBc-secreting B-lymphocytes. Anti-HBc may be a strong indicator for liver damage during a certain cellular immune response in CHB patients, and further studies on the mechanism of anti-HBc involvement hepatocellular injury will be worthwhile.

In HBeAg (+) CHB patient with normal ALT levels, 81.63% of the patients with moderate-to-severe inflammation were correctly identified using anti-HBc specific low cutoff value of 4.47 log_10_ IU/mL (which was established by maximizing the sum of sensitivity and specificity). And using anti-HBc specific high cut-off of 4.67 log_10_ IU/mL (which established by obtaining a specificity of at least 95%), 95.24% of the patients with mild or no inflammation (PPV 85.71%) were correctly excluded. In HBeAg (+) patient with normal ALT levels, using anti-HBc specific low cut-off value of 4.47 log_10_ IU/mL, 67.47% of the patients with moderate-to-severe inflammation were correctly distinguished. 95% of the patients with mild or no inflammation (PPV 75%) were correctly excluded using the cut-off of 5.00 log_10_ IU/mL. Therefore, to evaluate liver inflammation caused by HBV infection, the measurement of serum anti-HBc levels may provide accurate evidence to determine the antiviral choice in patients with normal ALT levels.

In conclusion, we are the first to demonstrate that serum anti-HBc is significantly corrected with hepatic inflammation in CHB patients with normal ALT. Serum anti-HBc is a promising noninvasive clinical biomarker that exhibits a high diagnostic accuracy for moderate-to-severe hepatic inflammation in patients with normal ALT levels.

## Patients and Methods

### Patients

A total of 655 consecutive treatment naïve patients with chronic HBV infection who underwent liver biopsy in 24 teaching hospitals located in mainland of China were enrolled into this study between October 2013 and February 2016. Patients recruited in the cohort study met the following criteria: (1) age 18–75 years; (2) HBsAg seropositive status beyond 6 months; (3) treatment naïve; (4) negative serum levels of anti-HAV IgM, anti-HCV, anti-HEV IgM/IgG, anti-EBV IgM, and anti-CMV IgM; and (5) off potential transaminase-lowering agents such as bicyclol for at least 2 weeks prior to blood sampling biochemistries. Exclusion criteria for this study included overlapping etiologies for hepatitis including HCV, human immunodeficiency virus (HIV), alcohol abuse, autoimmune, genetic, drug-induced and nonalcoholic fatty liver disease. Patients with decompensated cirrhosis or hepatocellular carcinoma were also excluded.

Additionally, of the aforementioned 655 patients, 45 CHB patients who underwent second liver biopsies after 78 weeks of antiviral-treatment were also enrolled.

All patents provided written informed consent for the scientific use of their clinical data and sample. The study was approved by the local ethics committee of Peking University First Hospital, and all methods were performed in accordance with the relevant guidelines and regulations. The complete protocol for the clinical trial has been registered at clinicaltrials.gov (NCT01962155) and chictr.org (ChiCTR-DDT-13003724).

### Histologic staging

Ultrasonographic-guided liver biopsies with a length ≥20 mm were routinely performed on all patients according to a standardized protocol after receiving the patient’s written informed consent. Pathological assessments were conducted in the Department of Pathology at the You-An Hospital affiliated with Capital Medical University. Each section was blindly and independently assessed by 2 pathologists. When discrepancies occurred, the samples were reviewed by experienced pathologists who were also responsible for reassessment in a randomly selected 10% of samples^26^. Disease activity grade was staged using the modified histology activity index (HAI), and hepatic fibrosis was assessed using the Ishak fibrosis score. For analysis, HAI ≥ 5 was considered moderate-to-severe inflammation, and F ≥ 3 was considered significant fibrosis^27, 28^.

### Laboratory measurements

At the time of liver biopsy, biochemical tests, blood cell and coagulation tests were performed using routine automated analyzers. Serum levels of hepatitis B surface antigen (HBsAg) and hepatitis B e (HBeAb) were quantified using commercially available enzyme immunoassays (Roche Diagnostics, Penzberg, Germany). Serum HBV DNA levels (range 2.0 × 10^1^–1.7 × 10^8^ IU/ml) were measured via a COBAS AmpliPrep/COBAS TaqMan method as previously described^29^.

### Anti-HBc measurement

Sandwich enzyme-linked immunosorbent assays for serum anti-HBc level were performed as described previously^30^.

### Statistical analysis

All statistical analyses were performed with SPSS ver.16.0 (Chicago, IL, USA). Quantitative variables were expressed as the mean ± standard deviation (SD). The chi-square test was used to analyze relationships between categorical variables, and Student’s *t* test was used to analyze single specific differences of biological interest. Spearman’s rank tests were used to analyze associations between variables and stages of hepatic pathology. A logistic regression was performed to analyze whether anti-HBc was an independent risk factor for significant inflammation in CHB patients.

The diagnostic abilities of anti-HBc and combination between anti-HBc and different variables were evaluated based on the estimated ROC curves and by calculations of the sensitivity, specificity, positive predictive values (PPV) and negative predictive value (NPV) for cut-off values. Statistical significance was defined as *P* < 0.05 (two-tailed).

## Electronic supplementary material

Supplementary Information
