# Supplementary material for: Serum hepatitis B core antibody as a biomarker of hepatic inflammation in chronic hepatitis B patients with normal alanine aminotransferase
Source: Sci Rep. 2017 Jun 5;7:2747. doi: 10.1038/s41598-017-03102-3 (PMC5459818; doi:10.1038/s41598-017-03102-3)
Supplement: Supplementary file 1 — Supplementary Information [file 41598_2017_3102_MOESM1_ESM.pdf]

Serum hepatitis B core antibody as a biomarker of hepatic inflammation in chronic hepatitis B patients with normal alanine aminotransferase

Jiyuan Zhou[1], Liuwei Song[2], Hong Zhao[1], Linlin Yan[1], Anlin Ma[3], Shibin Xie[4], Xuqing Zhang[5], Dazhi Zhang[6], Qing Xie[7], Guo Zhang[8], Jia Shang[9], Jun Cheng[10], Weifeng Zhao[11], Zhiqiang Zou[12], Mingxiang Zhang[13], Ningshao Xia[2,#], Guiqiang Wang[1,14,#]

1. Department of Infectious Disease, Center for Liver Disease, Peking University First Hospital, Beijing
2. State Key Laboratory of Molecular Vaccinology and Molecular Diagnostics, National Institute of Diagnostics and Vaccine Development in Infectious Diseases, School of Public Health, Xiamen University, Xiamen, China
3. Department of Infectious Disease, China-Japan Friendship Hospital, Beijing, China
4. Department of Infectious Disease, The Third Affiliated Hospital Sun Yat-Sen University, Guangzhou, Guangdong, China
5. Department of Infectious Diseases, South West Hospital affiliated to Third Military Medical University, Chongqing, China
6. Department of Infectious Diseases, Second Affiliated Hospital of Chongqing Medical University, Chongqing, China
7. Department of Infectious Diseases, Rui Jin Hospital Shanghai Jiao Tong University School of Medicine, Shanghai, China
8. Department of Infectious Diseases, The People's Hospital of Guang Xi Zhuang Autonomous Region, Nanning, Guangxi, China
9. Department of Infectious Diseases, The People's Hospital of He Nan Province, Zhengzhou, Henan, China
10. Department of Infectious Diseases, Di Tan Hospital affiliated to Capital Medical University, Beijing, China
11. Department of Infectious Diseases, Xinxiang Medical University Third Hospital, Xinxiang, Henan, China
12. Department of Infectious Diseases, Yan tai City Hospital for Infectious Disease, Yan tai, Shandong, China

13. Department of Infectious Diseases, Shenyang Sixth People's Hospital, Shenyang, Liaoning, China
14. Collaborative Innovation Center for Diagnosis and Treatment of Infectious Diseases, Zhejiang University, Hangzhou, Zhejiang, China

Supplementary Figure S1 Dynamic changes of liver fibrosis score in HBeAg (+)(**a**) and HBeAg (-)(**b**) CHB patients receiving antiviral treatment after a second liver biopsy.

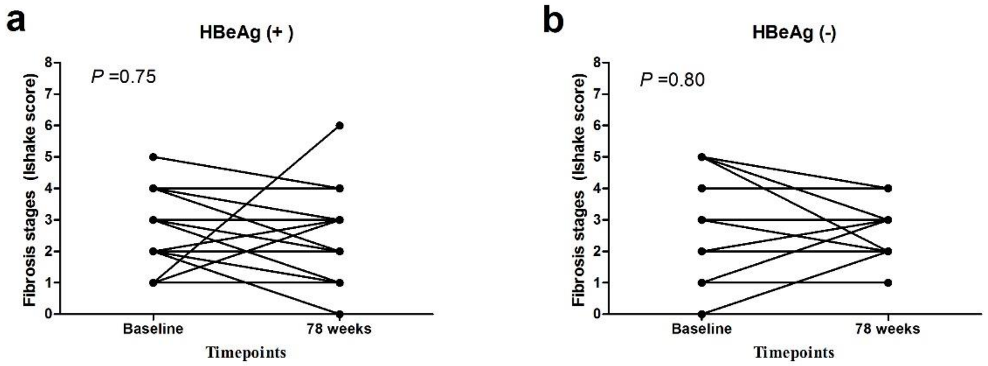

Supplementary Table S2 Univariate and multivariate analysis of clinical parameters, anti-HBc with liver inflammation in HBeAg (+) patients (n=404)

**Parameters**	**Univariate analysis**		***P* Value**	**Multivariate analysis**	***P* Value**
	**HAI <5 (n=155)**	**HAI ⩾5 (n=249)**	**(Univariate)**	**Odds Ratio (95% CI)**	**(Multivariate)**
Sex distribution, males	80% (124/155)	79.5% (198/249)	1		
age,years	36.03±10.18	26.51±10.45	0.53		
BMI(kg/m^2)	25.04±23.09	23.23±3.66	0.78		
HBsAg (log_{10} IU/ml)	4.08±0.92	4.61±0.58	< 0.001	0.83 (0.55-1.26)	0.38
HBV DNA (log_{10} IU/ml)	7.22±1.70	6.76±1.60	< 0.001	0.88 (0.72-1.07)	0.2
Anti-HBc (log_{10} IU/ml)	3.81±1.02	4.79±0.64	< 0.001	4.52 (2.54-8.06)	< 0.001
ALT (U/L)	56.08±59.66	137.6±163.4	< 0.001	1.00(0.99-1.02)	0.52
AST (U/L)	35.96±25.98	83.74±93.57	< 0.001	1.01 (0.99-1.03)	0.16
ALP (U/L)	73.33±23.58	86.36±28.96	< 0.001	1.00 (0.98-1.01)	0.48
GGT (U/L)	35.16±31.41	67.65±68.21	< 0.001	1.00 (1.00-1.01)	0.74
Tbil	14.79±18.01	17.68±13.57	< 0.001	1.00 (0.98-1.02)	0.97
Albumin	44.61±3.86	43.18±5.73	< 0.001	1.00 (0.93-1.07)	0.94
PT s	12.48±1.12	13.01±1.65	< 0.001	0.92 (0.66-1.29)	0.63
PTA %	101.4±18.44	94.29±16.03	0.001	1.00 (0.98-1.03)	0.93
INR	1.01±0.09	1.07±0.14	< 0.001	1.00 (0.98-1.02)	0.61
Platelet counts ($x10^9$/L)	190.7±58.72	171.3±53.15	< 0.001	1.00 (0.99-1.00)	0.43

HAI, histology activity index; Fibrosis Score; MBI, body mass index; HBsAg, hepatitis B surface antigen; HBeAg, hepatitis B e antigen; anti-HBc, hepatitis B core antibody; ALT, alanine aminotransferase; AST, aspartate aminotransferase; ALP, alkaline phosphatase; GGT, gamma-glutamyl transpeptidase; Tbil, total bilirubin; PT, prothrombin time; PTA, prothrombin time activity; INR, international normalized ratio.

Supplementary Table S3 Univariate and multivariate analysis of clinical parameters, anti-HBc with liver inflammation in HBeAg (-) patients (n=251)

**Parameters**	**Univariate analysis**		***P* Value**	**Multivariate analysis**	***P* Value**
	**HAI <5 (n=110)**	**HAI ≥5 (n=141)**	**(Univariate)**	**Odds Ratio (95% CI)**	**(Multivariate)**
Sex distribution, males	72.7% (80/110)	78.7% (111/141)	0.3		
age,years	41.42±9.78	41.87±9.56	0.71		
BMI(kg/m^2)	23.37±2.81	23.83±2.85	0.19		
HBsAg ($\log_{10}$ IU/ml)	0.78±0.07	4.67±0.58	< 0.001	1.32 (0.75-2.35)	0.34
HBV DNA ($\log_{10}$ IU/ml)	4.50±1.44	5.08±1.74	0.002	1.12 (0.85-1.46)	0.43
Anti-HBc ($\log_{10}$ IU/ml)	3.81±1.02	4.79±0.64	< 0.001	2.97 (1.38-6.39)	0.005
ALT (U/L)	45.29±30.64	108.7±132.5	< 0.001	1.00 (0.99-1.02)	0.97
AST (U/L)	35.45±22.63	73.63±88.26	< 0.001	1.02 (0.98-1.05)	0.33
ALP (U/L)	76.94±23.68	87.68±31.40	0.007	1.00 (0.99-1.02)	0.68
GGT (U/L)	31.57±27.46	70.55±65.73	< 0.001	1.03 (1.01-1.06)	0.01
Tbil	15.82±7.89	20.62±35.85	< 0.001	1.00 (0.98-1.02)	0.84
Albumin	45.79±5.44	44.21±6.46	0.004	0.96 (0.90-1.02)	0.2
PT s	12.40±1.32	12.68±1.60	0.25	0.83 (0.57-1.23)	0.36
PTA %	98.58±18.37	94.79±15.07	0.09	1.00 (0.98-1.03)	0.78
INR	1.01±0.09	1.04±0.12	0.09	1.00 (0.99-1.02)	0.14
Platelet counts ($x10^9$/L)	162.4±53.26	155.4±56.77	0.18	1.01 (1.00-1.01)	0.2

HAI, histology activity index; Fibrosis Score; MBI, body mass index; HBsAg, hepatitis B surface antigen; HBeAg, hepatitis B e antigen; anti-HBc, hepatitis B core antibody; ALT, alanine aminotransferase; AST, aspartate aminotransferase; ALP, alkaline phosphatase; GGT, gamma-glutamyl transpeptidase; Tbil, total bilirubin; PT, prothrombin time; PTA, prothrombin time activity; INR, international normalized ratio.

Supplementary Table S4 Univariate and multivariate analysis of clinical parameters, anti-HBc with liver fibrosis in HBeAg (+) Patients (n=404)

**Parameters**	**Univariate analysis**		***P* Value (Univariate)**	**Multivariate analysis**	***P* Value (Multivariate)**
	**F <3 (n=256**	**F⩾3 (n=148)**		**Odds Ratio (95% CI)**	
Sex distribution, males	80.9% (207/256)	77.7% (115/148)	0.44		
age,years	34.49±9.55	39.47±10.90	< 0.001	1.03 (1.00-1.06)	0.04
BMI(kg/m^2)	23.08±4.24	23.54±2.88	0.03	1.00 (0.98-1.02)	0.8
HBsAg (log_{10} IU/ml)	4.07±0.79	3.42±0.68	< 0.001	0.58 (0.37-0.91)	0.02
HBV DNA (log_{10} IU/ml)	7.22±1.63	6.46±1.60	< 0.001	0.84 (0.70-1.02)	0.09
Anti-HBc (log_{10} IU/ml)	4.20±0.98	4.48±0.75	0.005	1.30 (0.83-2.04	0.25
ALT (U/L)	105.1±122.5	108.6±164.5	0.76		
AST (U/L)	61.02±63.72	72.93±99.16	0.003	1.00 (0.99-1.00)	0.04
ALP (U/L)	77.39±24.96	80.06±30.92	0.001	1.00 (0.99-1.01)	0.8
GGT (U/L)	45.95±53.74	71.70±64.54	< 0.001	1.00 (1.00-1.01)	0.12
Tbil	15.97±18.02	17.61±9.52	< 0.001	1.00 (0.98-1.02)	0.94
Albumin	44.68±4.77	42.06±5.35	< 0.001	0.92 (0.86-0.99)	0.02
PT s	12.48±1.17	13.37±1.80	< 0.001	1.22 (0.86-1.75)	0.27
PTA %	101.0±16.31	90.49±17.01	< 0.001	1.00 (0.97-1.03)	0.81
INR	1.01±0.09	1.09±0.16	< 0.001	1.00 (0.99-1.01)	0.44
Platelet counts ($x10^9/L$)	193.3±52.64	153.6±52.98	< 0.001	0.99 (0.98-1.00)	<0.001

F, Fibrosis Score; MBI, body mass index; HBsAg, hepatitis B surface antigen; HBeAg, hepatitis B e antigen; anti-HBc, hepatitis B core antibody; ALT, alanine aminotransferase; AST, aspartate aminotransferase; ALP, alkaline phosphatase; GGT, gamma-glutamyl transpeptidase; Tbil, total bilirubin; PT, prothrombin time; PTA, prothrombin time activity; INR, international normalized ratio.

Supplementary Table S5. Univariate and multivariate analysis of clinical parameters, anti-HBc with liver fibrosis in HBeAg (-) Patients (n=251)

**Parameters**	**Univariate analysis**		***P* Value (Univariate)**	**Multivariate analysis**	***P* Value (Multivariate)**
	**F <3 (n=149)**	**F⩾3 (n=102)**		**Odds Ratio (95% CI)**	
Sex distribution, males	72.5% (108/149)	81.4% (83/102)	0.13		
age,years	40.81±9.63	42.90±9.58	0.11		
BMI(kg/m^2)	23.41±2.81	23.93±2.86	0.17		
HBsAg (log_{10} IU/ml)	3.19±0.71	3.10±0.75	0.3		
HBV DNA (log_{10} IU/ml)	4.82±1.60	4.83±1.7	0.86		
Anti-HBc (log_{10} IU/ml)	4.39±0.57	4.59±0.64	0.002	1.44 (0.78-2.65)	0.25
ALT (U/L)	67.83±78.35	98.82±133.8	0.008	1.00 (0.99-1.01)	0.49
AST (U/L)	45.17±47.60	73.19±91.01	< 0.001	1.01 (0.99-1.02)	0.43
ALP (U/L)	76.08±20.89	92.43±34.83	< 0.001	1.01 (1.00-1.03)	0.08
GGT (U/L)	38.72±38.87	73.33±68.16	< 0.001	1.01 (1.00-1.02)	0.03
Tbil	15.41±7.54	22.90±41.35	0.03	1.01 (0.98-1.04)	0.6
Albumin	45.58±4.92	43.96±7.33	< 0.001	0.98 (0.92-1.04)	0.47
PT s	12.36±1.39	12.83±1.58	0.01	1.03 (0.71-1.49)	0.88
PTA %	98.51±16.93	93.51±15.92	0.003	1.01 (0.98-1.04)	0.64
INR	1.01±0.10	1.05±0.11	0.002	1.00 (0.98-1.02)	0.62
Platelet counts ($x10^9$/L)	172.6±54.60	138.6±49.99	< 0.001	0.99 (0.98-0.99)	<0.001

F, Fibrosis Score; MBI, body mass index; HBsAg, hepatitis B surface antigen; HBeAg, hepatitis B e antigen; anti-HBc, hepatitis B core antibody; ALT, alanine aminotransferase; AST, aspartate aminotransferase; ALP, alkaline phosphatase; GGT, gamma-glutamyl transpeptidase; Tbil, total bilirubin; PT, prothrombin time; PTA, prothrombin time activity; INR, international normalized ratio.
